# Aromatic Plants and Their Associated Arbuscular Mycorrhizal Fungi Outcompete *Tuber melanosporum* in Compatibility Assays with Truffle-Oaks

**DOI:** 10.3390/biology12040628

**Published:** 2023-04-20

**Authors:** Vasiliki Barou, Ana Rincón, Cinta Calvet, Amelia Camprubí, Javier Parladé

**Affiliations:** 1 Centre de Cabrils, Institut de Recerca i Tecnologia Agroalimentàries, IRTA, Ctra. Cabrils km. 2, E-08348 Cabrils, Spain; cinta.calvet@irta.cat (C.C.); amelia.camprubi@irta.cat (A.C.); 2 Instituto de Ciencias Agrarias, ICA-CSIC, C/Serrano 115 dpdo., E-28006 Madrid, Spain; ana.rincon@csic.es

**Keywords:** *Tuber melanosporum*, truffle-oaks, aromatic plants, arbuscular fungi, mycorrhizas, intercropping, competition, lavender, thyme, sage

## Abstract

**Simple Summary:**

Truffle culture is a fairly profitable agricultural practice, yet there is a long waiting period to reach peak sporocarp production from the point when ectomycorrhizal truffle-oak seedlings are planted in the field. Adding a secondary crop, such as medicinal and aromatic plants, could enhance the sustainability of truffle agro-forest systems. In this work, we study the relationships between oaks and aromatic plants and their associated mycorrhizal fungi (either ectomycorrhizal or arbuscular mycorrhizal, respectively) under controlled conditions. A reciprocal competition effect is revealed between oaks and aromatic plant species, as well as between the different types of mycorrhizal fungi. Our results indicate that managing arbuscular mycorrhizal fungi in truffle plantations is a relevant factor to be considered when establishing the dual cultures of plant species and mycorrhizal types in intercropping systems.

**Abstract:**

The high value of black truffle recompenses the slow growth of the fungus when established in the field. Adding a secondary crop, such as medicinal and aromatic plants (MAPs), could further enhance the sustainability of truffle production agro-forest systems. The dual cultures of ectomycorrhizal truffle-oak seedlings and MAPs (lavender, thyme, and sage) previously inoculated and non-inoculated with native arbuscular mycorrhizal fungi (AMF), were established to evaluate plant–fungi relationships. After 12 months in a shadehouse, plants’ growth, mycorrhizal colonization, and extraradical soil mycelium (both of *Tuber melanosporum* and AMF) were measured. Overall, truffle-oaks’ growth was negatively affected by the presence of MAPs, especially when inoculated with AMF. In turn, the presence of truffle-oaks barely affected the co-cultured MAPs, and only lavenders showed a significant growth reduction. All AMF-inoculated MAPs showed higher shoot and root biomass than non-inoculated ones. Compared to truffle-oaks growing alone, the presence of co-cultured MAPs, especially when they were AMF-inoculated, significantly decreased both the ectomycorrhizas and soil mycelium of *T. melanosporum*. These results reveal the strong competition between AMF and *T. melanosporum* and warn about the need for the protection of intercropping plants and their associated symbiotic fungi to avoid reciprocal counterproductive effects in mixed truffle-oak–AMF–MAP plantations.

## 1. Introduction

Truffles are edible ectomycorrhizal fungi greatly appreciated and widely used in international haute cuisine [1]. Like other truffle species, the black truffle (*Tuber melanosporum* Vittad.) forms mycorrhizal associations with the roots of diverse hosts, such as the *Quercus* species, to complete its lifecycle and produce edible fruitbodies [2]. During the twentieth century, the production of the black truffle moved from woodlands to planted truffle orchards [3], playing an economic, cultural, and structural role in Mediterranean landscapes [4]. Truffle production in truffle orchards emerges a minimum of five years after the trees are planted, and it takes from seven to eleven years to achieve peak production [1]. Thus, intercropping strategies, i.e., the agronomic practices of growing two or more compatible crops simultaneously on the same field, could allow truffle growers to integrate a secondary crop in pre-productive truffle plantations to improve the ecosystem services and profitability. Compared to monocultures, diverse agroecosystems created by intercropping generate remarkable benefits, such as increased biodiversity, enhanced pest control, and erosion prevention, as well as diversified economic yields [5]. Moreover, this traditional farming system enhances not only crop productivity but also the efficient utilization of resources, both above- and below-ground [6,7]. Intercrops should be designed to take maximum advantage of their potential for complementarity and the facilitation of the involved crops, considering the choice of species or variety, input levels, sowing dates, and spatial–temporal configuration [8].

Intercropping with medicinal and aromatic plants (MAPs) can increase the stability of agroforest ecosystems [9]. For example, intercropping with basil (*Ocimum basilicum* L.) and summer savory (*Satureja hortensis* L.) significantly increased soil organic nitrogen and available nitrogen contents, improving soil quality in orchard ecosystems [10]. A recent survey in Southern France indicated that truffle growers relate intercropping with MAPs to the improvement of truffle production and soil organic matter and, to the enhanced drought resilience of truffle orchards [11,12]. Among compatible crops, MAPs are a suitable option to combine with Mediterranean truffle plantations, since they are adapted to the same ecological conditions as truffle-oaks. However, truffle brûlés, the areas with scarce vegetation due to the allelopathic effects of truffle mycelium in the soil, could limit intercropping possibilities by reducing plant growth and associated soil microbiota [13].

It is necessary to understand the ecological processes underlying intercropping designs, such as the nature of the relationships between plants and mycorrhizal fungi, to reach an outcome that increases sustainability and ecosystem services [6]. Interactions between plants and microbes benefit plants by increasing the acquisition of nutrients, producing growth hormones [14], and defending against diseases and pests [15]. More specifically, in MAPs, previous studies have shown an increased yield of shoot biomass and essential oil when sage (*Salvia officinalis* L.), thyme (*Thymus vulgaris* L.), and oregano (*Origanum vulgare* L.) plants were inoculated with arbuscular mycorrhizal fungi (AMF) [16]. In the case of coriander (*Coriander sativum* L.), the AMF inoculation served as a bio-fertilizer, promoting the growth and nutrient acquisition of plants [17].

Few studies have shown the effects of interactions between truffle plants (forming ectomycorrhizas, ECM) and shrubs and herbs (companion plants, mostly forming arbuscular mycorrhizas, AM) either cultivated or growing spontaneously in truffle brûlés [18,19]. Previous studies on AM–ECM fungal interactions were mostly conducted at the scale of plant individuals hosting multiple types of mycorrhizal fungi [20,21]. Among them, plants of the genus *Quercus* are confirmed to form both kinds of mycorrhizal symbioses, forming AM and ECM structures [22]. However, the simultaneous reciprocal interaction of plants hosting AM and ECM fungi has not yet been sufficiently explored [23]. Knoblochová et al. [24] studied the co-occurrence of the AM grass *Calamagrostis epigejos* Roth (L.) and the predominantly ECM tree *Salix caprea* L. at post-mining sites that were spontaneously colonized by vegetation. Aside from the microenvironmental effects on both fungal communities, the presence of *S. caprea* significantly decreased AM fungal abundance in soil as well as AMF colonization and richness in *C. epigejos* roots. McHugh and Gehring [25] observed the opposite trends in semi-arid, pine–juniper woodland, where the density of the AM shrub *Juniperus monosperma* (Engelm.) Sarg. was negatively correlated with the ECM colonization of *Pinus edulis* Engelm. and ECM fungal abundance in soil [26].

More recently, Taschen et al. [19] showed that a selection of companion plants, all of them hosting AMF, promoted the development of truffle mycelium in a rhizotron experiment established with evergreen oaks (*Quercus ilex* L.), growing together with different species of AM plants. Glomeromycotina (AMF) mycelia in soils inoculated with *T. melanosporum* was six times lower than in non-inoculated rhizotrons. However, the experimental AMF inoculation of companion plants was not included in this assay and, consequently, not controlled. In our study, we designed a mesocosm experiment [27] to evaluate the interactions between truffle-inoculated evergreen oak seedlings and three species of MAPs, namely lavender (*Lavandula officinalis* Chaix.), thyme, and sage, either non-inoculated or inoculated with native AMF. We hypothesize that: (i) the growth of co-cultured truffle-oaks and MAPs will be reduced as compared to monocultures; (ii) in the case of MAPs, this negative effect of co-culture will be reduced by mycorrhization with native AMF; and (iii) *T. melanosporum* colonization (ectomycorrhizas and extraradical mycelium) will outcompete AMF in dual cultures. 

## 2. Materials and Methods

### 2.1. Experimental Setup, Plant Material, and AM Inoculum Production

A mesocosm experiment was conducted during a period of one year in a shadehouse with open ends located at the Institute of Agrifood Research and Technology (IRTA) in Cabrils, Barcelona (41°30′58.6″ N, 2°22′36.7″ E). The area has a Mediterranean climate with a mean annual rainfall of 614 mm and a mean annual temperature of 16 °C. 

One-year-old evergreen oaks inoculated with spores of *T. melanosporum* were obtained in June 2020 from a commercial nursery (‘Viveros Alto Palancia’, Castellón, Spain). The seed provenance was ‘Sistema Ibérico’, lot 12/1049_OR/2019/A001/00002. The seedlings were originally produced in 650 cc containers filled with a commercial substrate containing peat, perlite, and disinfected soil. Ten seedlings were randomly selected for visual examination under a stereomicroscope for truffle ectomycorrhizal colonization before establishing the experiment. The initial percentage of ectomycorrhizas (number of ECM tips/total number of, at least, 200 short roots) was around 20.6 ± 9.3 (SD). Plantlets of lavender, thyme, and sage produced in peat plugs were obtained from a commercial nursery (Aldrofeu and Associats, Riudarenes, Girona, Spain) in April 2020.

Native AM inoculum was isolated from different weeds growing inside the brûlés of three productive truffle plantations in Spain: Batea (Tarragona), La Matilla (Segovia), and Montan de Tost (Lleida). Different AM plant species were carefully collected in June 2019 from the brûlés. The rhizosferic soil attached to the plants’ roots from each site was added into 250 cc pots filled with sterilized sand (120 °C, 1 atm, 1 h) from a quarry. A trap plant (*Allium porrum* L.) was then transplanted into each pot to recover the AM inoculum as described in Camprubí and Calvet [28]. The final AM inoculum consisted of a mixture of colonized roots, spores, and extraradical mycelium in a matrix of sand. The inocula from the three sites were examined under the microscope to identify the presence of AMF propagules, and all of them displayed *Glomus*-type spores (Figure S1a). The three AM inocula were mixed in the same volume, and half of the MAPs were inoculated with 10 mL of AMF mixed inoculum in 250 cc individual pots filled with the same sand, while the other half were transplanted but remained non-inoculated. In July 2020, the MAPs were assessed for AM root colonization using the gridline intersection method [29] and were then ready for transplantation.

### 2.2. Experimental Design and Management

**Figure 1:**
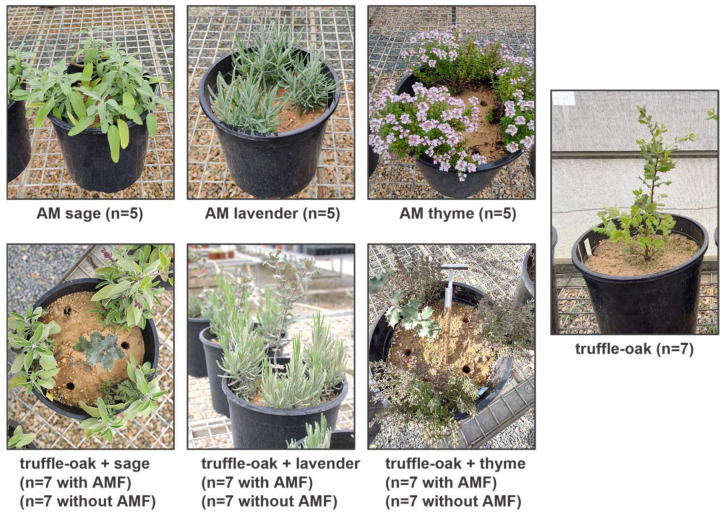
Experimental set-up of interactions between evergreen oak ectomycorrhizal with *Tuber melanosporum* (truffle-oak) and three species of medicinal and aromatic plants (MAPs), inoculated with arbuscular mycorrhizal fungi (AMF) or non-inoculated. Truffle-oak and MAPs growing alone were used as controls.

In June 2020, topsoil up to 30 cm depth was collected in a field next to a productive *T. melanosporum* plantation, free of potential host plants, in Batea (Tarragona, Spain). The soil was mechanically mixed with 10% perlite, distributed into 10 L plastic bags, and autoclaved (120 °C, 1 atm, 1 h). The soil was analyzed after sterilization (Table S1). The physico-chemical soil analysis showed normal or non-limiting parameters for a truffle producing area [30] with a low organic matter and phosphorus and high calcium content. Sixty-four containers (25 cm diameter, 8 L capacity each) were filled with the autoclaved soil–perlite mixture. One truffle-oak seedling was transplanted into each container and three MAPs (either sage, lavender, or thyme), either mycorrhized with AMF or not, were transplanted around each oak next to the edge of the container to study dual plant and fungal interactions (Figure 1). Control monoculture treatments with either AM–MAPs or truffle-oak seedlings growing alone were also included in the design.

The experiment was established in July 2020 and maintained under regular watering in the shadehouse for 12 months. No fertilizers were applied throughout the experiment. The positions of the containers on the bench were rotated once a month. In the middle of the experiment (December 2020), MAPs were partially pruned to avoid physical competition with oaks.

### 2.3. Plants Growth and Physiological Parameters

At the end of the experiment, in July 2021, we measured the height of all plants and the diameter of the oaks, as well as counting the number of spikes of lavenders. We also measured the leaf mass per area (LMA) and the chlorophyll content of new leaves with a SPAD-502, (Konika Minolta Sensing, Inc., Tokyo, Japan). Plants were then harvested and root and shoot biomass were measured after air-drying at 65 °C for 48 h in a drying oven (UF1060, Memmert GmbH & Co. KG, Schwabach, Germany). The dry weight of MAPs was averaged for each container after adding the dry weight of the middle-season pruning.

### 2.4. Quantification of Mycorrhizas

The percentage of the ECM of *T. melanosporum* in oak plants at the end of the experiment [(No. mycorrhizas/No. of total short roots) × 100] was calculated from a minimum of 200 short roots of each plant examined under a stereomicroscope. A sample of roots of the MAPs was clarified and stained [31] and the percentage of cortex infected by the AMF was estimated visually under a stereomicroscope, as described in Giovannetti and Mosse [29].

### 2.5. Molecular Analyses of Soil Samples

Soil cores were taken at three points, equidistant between the MAPs and the center of each container, by using a 15 cm-long cheese probe (Figure 1) and then pooled into one composite sample per container. Soil samples were sieved with a 2 mm mesh and dried at 50 °C in a heater with recirculating air. Then, we extracted total DNA from 0.25 g of each soil sample using the DNeasy Power Soil HTP 96 kit (Qiagen, Hilden, Germany). The concentration of the yielded DNA per sample was determined with a Qubit 2.0 Fluorometer (Thermo Fisher Scientific, Wilmington, DE, USA). The extracted DNA was stored at −20 °C until further analysis. 

The extraradical mycelium biomass of *T. melanosporum* from each soil sample was quantified by real-time Taqman^®^ PCR (qPCR) in a StepOnePlus™ Real-Time PCR System (Applied Biosystems™ by Thermo Fisher Scientific, Wilmington, DE, USA), as described in Parladé et al. [32]. The standard curve was created using DNA extracted from known gleba amounts of a fresh immature sporocarp [33]. Five ten-fold DNA dilutions were prepared to generate a standard curve from 40 to 0.0004 mg mycelium/g soil.

The soil biomass of AMF in the containers with MAPs and truffle-oaks was quantified by comparative qPCR-CT (ΔΔC_T_ method) using the same DNA extractions as described above. This method was originally designed to calculate the relative expression ratio of a target gene in different samples [34], using reference genes as internal reaction control to normalize mRNA levels between different samples. In our experiment, we used this method with the total soil DNA extractions to calculate the relative quantity between AM fungal mycelium in soils with AM–MAPs growing alone (controls) and soil samples from co-cultures (aromatic plants and oaks). The fold changes of AM fungal mycelium in the soil samples were calculated with the 2^−ΔΔCt^ formula (ΔC_T_ = C_T_ target gene − C_T_ reference gene; ΔΔC_T_ = ΔC_T_ target sample − ΔC_T_ reference sample). The primers AM1 [35] and AMG1F [36] were used to quantify Glomeromycota, as described in Bodenhausen et al. [37], with the same thermocycling conditions except for the annealing temperature, which was set at 60 °C. To normalize the samples to the same amount of total soil fungi, several genes were tested as endogenous controls (Table S2). We chose ITS1F [38] and ITS2 [39] primers, covering the ITS1 rDNA region of most fungi [40], because they were the only primers giving consistent amplification in all the soil samples. The relative amount of AMF mycelium and the total amount of fungi present in the samples was calculated separately for each plant species (lavender, sage, or thyme). 

To check the possible bias from using the multicopy ITS1 rDNA region as endogenous gene, we performed qPCR analyses from the DNA extractions of different soil samples, corresponding to different inoculation treatments, and adjusted accordingly in order to obtain the same amount of DNA (measured with a Qubit fluorometer). We obtained very close amplification curves from all the samples (Figure S2), indicating that the same fungal DNA concentrations from different samples showed similar C_T_ values.

### 2.6. Statistical Analyses

The data of plant growth, plant physiology, mycorrhizal colonization, and soil mycelial biomass were analyzed through analysis of variance (ANOVA), after checking for the normality and variance homogeneity of data. The shoot biomass of truffle-oaks was analyzed by ANCOVA to remove the influence of the initial height of the plants. When necessary, variables were log or log(x + 1) transformed to fit normal distribution. The percentages of *T. melanosporum* mycorrhizas were analyzed through generalized linear models considering the binary distribution of colonized vs. non-colonized roots. Soil mycelium colonization relationship between *T. melanosporum* and AMF was assessed by Spearman’s correlation analysis. 

All analyses were performed with the R software v. 4.2.1 [41], and figures with statistical data were produced using the plotly package [42]. 

## 3. Results

### 3.1. Truffle-Oaks and MAPs Development

**Figure 2:**
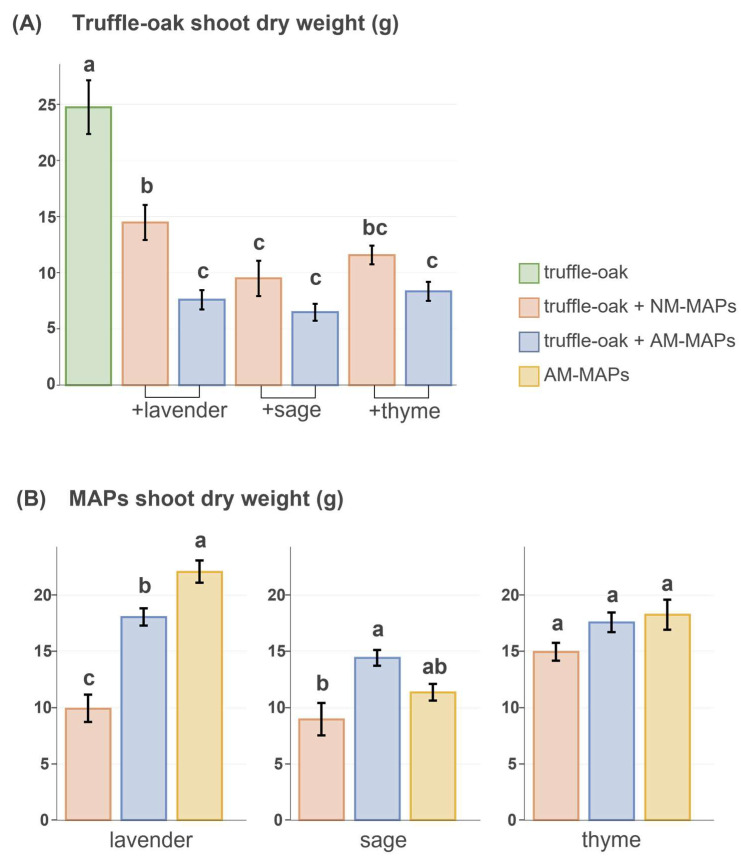
(**A**) Shoot dry weight of truffle-oak seedlings growing alone (*n* = 7, green) or in the presence of MAPs, either non-inoculated (NM, *n* = 7 per species, pink) or inoculated with AMF (AM, *n* = 7 per species, blue). (**B**) Shoot dry weight of lavender, sage, and thyme co-cultured with truffle-oaks and either non-inoculated (NM, pink) or inoculated with AMF (AM, blue), and growing alone (*n* = 5 per species, yellow). Data include means ± standard errors. Significant differences between means were analyzed by ANOVA for each MAP species separately and by ANCOVA in the case of truffle-oaks with the initial height as covariate. Treatments sharing the same lowercase letter are not significantly different (Tukey’s test, *p* < 0.05).

Overall, truffle-oak growth (shoot dry weight, height, diameter) and LMA were negatively affected by the presence of MAPs, particularly when these were inoculated with AMF (Figure 2A; Table S3). Root dry weight and chlorophyll content in oak leaves were unaffected by co-cultured MAPs (Table S3). Only in co-cultures did the two-way ANOVA analysis of truffle-oak shoot biomass, considering the initial height of the oaks as a covariate, show a significant effect of AMF inoculation on MAPs (F = 28.340, *p* < 0.001), of MAP species identity (F = 4.766, *p* = 0.015), and the interaction of the two (F = 3.994, *p* = 0.0274).

The presence of truffle-oaks affected MAPs differently (Figure 2B; Table S4). Only AM lavenders growing together with truffle-oaks had significantly less biomass than those growing alone. All the inoculated MAPs in a co-culture with truffle-oaks had significantly more shoot biomass than non-inoculated ones. The number of lavender spikes was significantly lower in non-inoculated plants growing together with truffle-oaks as compared to those inoculated with AM growing either in monoculture or co-culture with oaks (Table S4). 

### 3.2. Quantification of Tuber melanosporum

**Figure 3:**
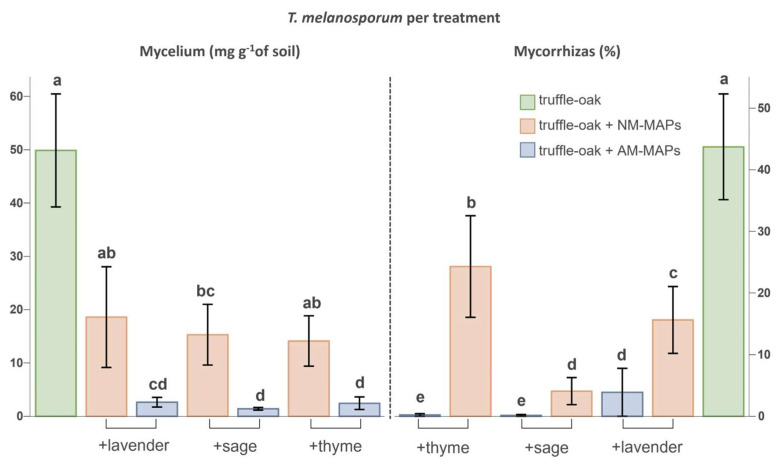
*Tuber melanosporum* extraradical mycelium biomass (graph on the left) and truffle ectomycorrhizal percentages of oaks (graph on the right). Truffle-oaks were growing alone (*n* = 7) or in presence of MAPs, either non-inoculated (NM, *n* = 7 per species) or inoculated (AM, *n* = 7 per species) with native AMF. Data include means and standard errors. Mycelium biomass was analyzed by one-way ANOVA (F_(6,42)_ = 12.65, *p* < 0.0001) and the percentages of ectomycorrhizas by generalized linear models (Chi-Square_(6,42)_ = 1466, *p* < 0.0001) assuming a binomial distribution of colonized vs. non-colonized roots. In each graph, treatments sharing the same lowercase letter are not significantly different (Tukey’s test, *p* < 0.05).

Truffle-oaks growing alone showed, on average, 44% ± 22.7 (SD) of *T. melanosporum* ectomycorrhizas (ECM) at the end of the experiment. This represents more than twice the initial percentage of ectomycorrhizas, indicating that the established mesocosms conditions were appropriate to allow the secondary colonization of the oak roots. The amount of extraradical mycelium and ectomycorrhizas of *T. melanosporum* drastically decreased in the presence of MAPs, especially of AM-inoculated MAPs (Figure 3). The percentage of *T. melanosporum* ECM was highly correlated to the soil mycelium biomass (Spearman, r = 0.7056, *p* < 0.0001). Both *T. melanosporum* mycorrhizas and extraradical mycelium were significantly correlated with truffle-oak biomass (Spearman, r = 0.6692 and r = 0.7555, respectively, *p* < 0.001).

### 3.3. AMF Quantification in MAPs and Truffle-Oaks

The relative quantification of AM fungal mycelium in soil revealed a significant effect of the presence of truffle-oaks on AMF. The AMF mycelium in soils with co-cultures of truffle-oaks and AM–MAPs was expressed by as much as 35–40% of their respective control treatments consisting of monocultures of each AM–MAP (Table S5). No significant differences in AMF mycelium reduction were found between co-cultured MAP species’ identities (ANOVA, F_(2,18)_ = 0.0393, *p* = 0.9616). The relative quantity of AMF mycelium in soil with the co-cultures of truffle-oak and AM–MAPs (i.e., the fold change relative to control AM–MAPs growing alone) was negatively correlated with the *T. melanosporum* mycelium biomass (Spearman, r = −0.5922, *p* = 0.0055), indicating a significant competition between extraradical mycelium of both types of mycorrhizal fungi. Conversely, the percentage of AMF root colonization was not significantly reduced in co-cultured MAPs, as compared to MAPs growing alone (Table S6). 

Truffle-oak seedlings growing together with AM–MAPs showed an elevated colonization by AMF (~80%) (Figure S3). Since no AM colonization was observed on oaks growing alone, the presence of AM colonization was derived, exclusively, from the companion AM–MAPs. No significant correlation was found between the percentage of AM mycorrhizas and truffle-oak biomass (Spearman, r = −0.1233, *p* = 0.5929).

## 4. Discussion

The plantation of medicinal and aromatic plants in truffle orchards is considered a convenient alternative to overcome the lack of benefits of early truffle plantations until sporocarp onset [11]. MAPs and oak trees establish different kinds of mycorrhizal associations, arbuscular mycorrhizas and ectomycorrhizas, respectively. On the other hand, truffle mycelium exerts a strong allelopathic effect that usually limits the growth of both plants and microorganisms in its influential area (the brûlé) [13]. This study was designed to evaluate the competition relationships between truffle mycelium (establishing ectomycorrhizas with oaks) and companion MAPs and their associated AMF under controlled conditions. 

The co-culture of young truffle-oaks and MAPs resulted in the growth reduction of truffle-oaks (especially the shoot dry weight) and the loss of ectomycorrhizas as compared to the control, and this effect was strengthened when the companion MAPs were inoculated with native AMF, supporting our first and second hypotheses. Notably, the essential oils of many MAPs contain volatile compounds with strong antifungal activity, especially against Ascomycetes [43,44,45,46,47,48]. Although this effect has not yet been tested on *Tuber* mycelium in the field, in vitro experiments have shown that these compounds inhibit its growth [49].

The growth reduction of the oaks could be explained by the competition for soil nutrients and the overall loss of *T. melanosporum* ectomycorrhizas. However, the effects of the ectomycorrhizas of *T. melanosporum* on the growth of *Quercus* seedlings is unclear. Taschen et al. [19] found out that inoculation of *Q. ilex* seedlings with *T. melanosporum* did not enhance development in height or basal diameter after three years’ growth in mesocosms conditions. On the other hand, Domínguez Nuñez et al. [50] observed a significant growth increase in *Q. ilex* seedlings inoculated with *T. melanosporum* spores as compared to non-inoculated ones after one year in the field. In our study, we detected a positive correlation of ectomycorrhizas and the extraradical mycelium of *T. melanosporum* with oak biomass, while no effect of AMF colonization on oak biomass was found. The chlorophyll content in *Q. ilex* leaves was unaffected by the presence of companion plants, either inoculated or non-inoculated. Our results agree with those of Taschen et al. [19]. The LMA values in oak leaves indicated that there was a higher competition for water in dual cultures as compared to oaks growing alone, but there were no significant differences among the treatments with and without inoculated AMF. Some studies in Mediterranean woody species and evergreen oak forests showed a negative correlation between the LMA and water availability of their habitat, i.e., the higher the LMA, the lower the water content in soil [51,52].

*Quercus* spp. are usually considered ectomycorrhizal, although AM fungal colonization seems to be also common, e.g., in section Lobatae (red oaks from North, Central, and South America) [53]. Dual mycorrhizal status is beneficial for the nutritional requirements of oaks in water-limited ecosystems [54] and as an insurance strategy in disturbed ecosystems when growing apart from other ectomycorrhizal trees [22]. Smith et al. [55] and Dickie et al. [53] found out that AMF infection in Pinaceae and *Q. rubra*, respectively, is most successful when a predominately AM host is present. Previous studies have suggested that the effects of AMF on *Quercus* spp. may be related to plant survival rather than growth effects [56]. We are not aware of other studies examining the ecology of AM fungal colonization and growth effects, specifically on the *Quercus* species of section *Ilex*. In our study, truffle-oaks growing alone did not show AM fungal colonization, whereas those growing together with arbuscular mycorrhizal MAPs showed an elevated root AM colonization (over 70%). This was coupled with a sharp reduction in truffle ectomycorrhizas, indicating both the strong competition of fungal mycorrhizal types and that dual *Q. ilex* mycorrhization is likely to occur in nature.

In our experimental conditions, MAPs also suffered from the competition of truffle-oaks, showing a significant decrease in growth and number of spikes (lavender), when comparing AM–MAPs growing alone with those growing together with truffle-oaks. González-Armada et al. [18] reported numerous vascular plants growing inside the truffle brûlés, both in plantations and wild areas, including the MAPs *Lavandula latifolia* Medic. and *Thymus vulgaris* L., despite the truffle’s allelopathy that inhibits the plant growth and coverage. However, most showed dwarfism and some were unable to complete their life cycle. Moreover, the diversity of ectomycorrhizal fungi and vascular plants were significantly reduced inside the brûlé.

Our results also show a protective effect of AMF in inoculated MAPs co-cultured with truffle-oaks, in which the former grew significantly more than the non-inoculated ones. This finding is consistent with numerous studies that have proven the beneficial effects of AMF on MAPs’ tolerance to abiotic stresses, plant biomass, and essential oil content [57,58,59,60,61,62]. Some of the intercropping experiences in truffle orchards in Southern France included co-cultivated lavender rows and showed strong competition with truffle production [11,12]. According to our results, lavender growth is strongly affected in the presence of *T. melanosporum*, but this effect can be significantly reduced when inoculated with AMF.

In our study, similar to that observed for truffle ectomycorrhizas, the presence of MAPs in dual cultures significantly lowered *T. melanosporum* mycelium in the soil, making it almost undetectable when the companion MAPs were inoculated with native AMF, which lead us to reject our third hypothesis. The results from molecular analyses also showed a significant negative correlation between AM fungal biomass and the mycelium of *T. melanosporum* in the soil. Taschen et al. [19] found out that the presence of some companion plants, such as *T. vulgaris* and *Festuca ovina* L., empirically associated by the orchard’s owners with better truffle production, had no particularly positive effect on *T. melanosporum* mycelium abundance in rhizotrons. However, the companion plants were not inoculated and the abundance of *T. melanosporum* mycelium in the soil of inoculated truffle-oaks at the end of the experiment was almost three times that obtained in our study.

The mesocosm conditions allowed us to establish replicated experiments while maintaining some of the key dimensions of the natural systems to be studied (soil, companion plants, truffle colonization, and AMF colonization), which allowed us to infer causal relationships from observable effects [63,64]. However, possible limitations derived from soil sterilization and the density restrains regarding plant growth [65] make it necessary to conduct further studies to confirm our obtained results under natural field conditions.

## 5. Conclusions

Co-culturing truffle-oaks and MAPs revealed strong interactions at both the plant and the fungal level under controlled conditions. Co-cultured truffle-oaks and MAPs, especially lavender, significantly reduced their growth as compared to their respective monocultures. However, this negative effect was buffered by their colonization with native AMF. In co-cultures, the AMF from MAPs likely competed with the *T. melanosporum* that was readily forming ectomycorrhizas with oaks, and in fact, a significant reduction in oak ectomycorrhizal percentage was observed. On the other hand, MAPs benefited from their associated arbuscular mycorrhizal fungi that were unaffected by co-culturing with oaks and promoted aromatic growth and, contrary to what was expected, also outcompeted the extraradical mycelium of *T. melanosporum* in soil. Overall, our results indicate that managing AMF in truffle plantations is an important factor to be considered when establishing the dual cultures of plant species and mycorrhizal types in intercropping systems.

## Data Availability

Not applicable.

## Supplementary Materials

The following supporting information can be downloaded at: https://www.mdpi.com/article/10.3390/biology12040628/s1, Figure S1: Stereomicroscope photographs of arbuscular mycorrhizal fungi (AMF) structures; Figure S2. Amplification plot of fungal ITS1 multicopy gene; Figure S3: *Quercus ilex* roots colonized by (a) *Tuber melanosporum* and (b) arbuscular mycorrhizal fungi, from co-cultured plants collected at the end of the experiment; Table S1: Physical-chemical properties of the soil used in the compatibility assays; Table S2: Primer pairs tested in this work; Table S3: Truffle-oak’s growth variables; Table S4: Medicinal and aromatic plants’ (MAPs) growth variables; Table S5: Quantification of arbuscular mycorrhizal (AM) extraradical mycelium in soils with truffle-oak growing together with AM lavender, sage, or thyme; Table S6: Percentage of arbuscular mycorrhizas (AM) in roots of MAPs growing alone or with truffle-oaks. References [66,67,68] are cited in here.

## Abbreviations

AM	Arbuscular mycorrhizal
AMF	Arbuscular mycorrhizal fungi
ECM	Ectomycorrhizal
LMA	Leaf mass per area
MAPs	Medicinal and aromatic plants
