# Supplementary material for: Aromatic Plants and Their Associated Arbuscular Mycorrhizal Fungi Outcompete Tuber melanosporum in Compatibility Assays with Truffle-Oaks
Source: Biology (Basel). 2023 Apr 20;12(4):628. doi: 10.3390/biology12040628 (PMC10136101; doi:10.3390/biology12040628)
Supplement: Supplementary file 1 [file biology-12-00628-s001.zip › biology-2329363-supplementary.pdf]

biology

MDPI

Supplementary Material

Aromatic plants and their associated arbuscular mycorrhizal fungi outcompete *Tuber melanosporum* in compatibility assays with truffle-oaks.

Vasiliki Barou, Ana Rincón, Cinta Calvet, Amelia Camprubí and Javier Parladé

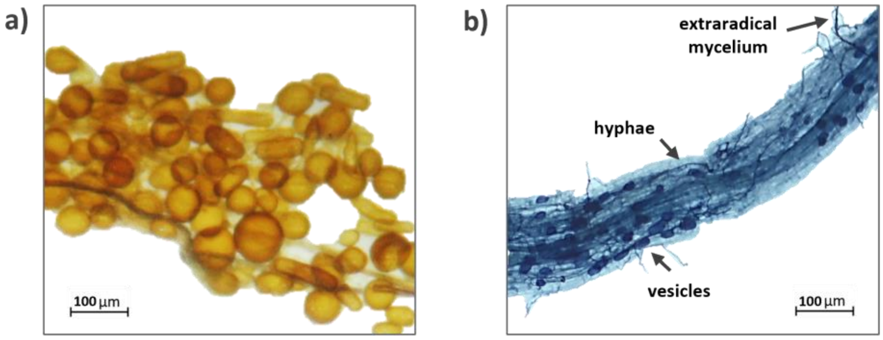

Figure S1. Stereomicroscope photographs of arbuscular mycorrhizal fungi (AMF) structures **(a)** Inoculum of *Glomus* spp-type spores isolated from native plants within the brûlés. (**b)** Root fragment of mycorrhizal lavender stained with trypan blue.

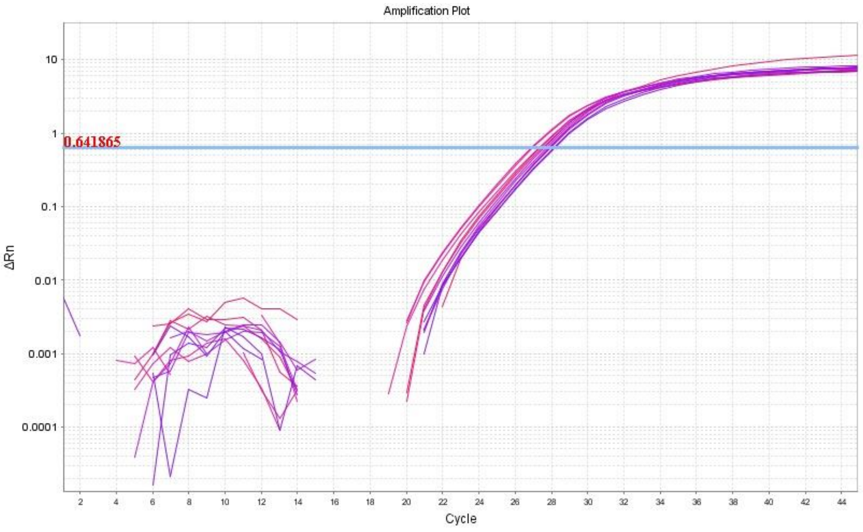

Figure S2. Amplification plot of fungal ITS1 multicopy gene. DNA samples from different treatments and different initial DNA concentrations were fitted to equal concentrations and then assessed in relative qPCR. Despite the unknown number of the initial ITS1 copies of each sample, the ITS1 gene was similarly expressed in all fitted samples. Mean C_T values in the established threshold (0.641865) was 27.56 ± 0.42 standard deviation.

Table S1. Physical-chemical properties of the soil used in the compatibility assays. Organic matter was measured by Walkey-Black method. Elements were determined by spectrophotometry, after ammonium acetate (*) or diethylenetriaminepentaacetic acid (DTPA) (‡) soil extraction. Texture was determined by the Bouyoucos method.

Analysis	Result
Gravimetric moisture 105°C	<1.000 %
pH (ext. 1:2.5 v: v, H_2O)	8.5
Electrical Conductivity 25°C (ext. 1:5 v: v, H_2O)	0.173 dS m^{-1}
Organic Matter	1.41 %
N-NO_3	12.9 mg kg^{-1}
P (Olsen)	9.8 mg kg^{-1}
K*	149 mg kg^{-1}
Ca*	7304 mg kg^{-1}g
Mg*	167 mg kg^{-1}
Na*	27 mg kg^{-1}
Fe‡	4.505 mg kg^{-1}
Mn‡	5.787 mg kg^{-1}
Zn‡	3.881 mg kg^{-1}
Cu‡	0.322 mg kg^{-1}
Al‡	0.317 mg kg^{-1}
Pb‡	3.256 mg kg^{-1}
Cd‡	0.018 mg kg^{-1}
Ni‡	0.125 mg kg^{-1}
B‡	0.037 mg kg^{-1}
Sand	52 %
Clay	21.4 %
Lime	26.60 %
Stoniness	58.2 %

Table S2. Primer pairs tested in this work: specific primers to amplify arbuscular mycorrhizal fungi (AMF) genes and generic fungal primers tested as endogenous control (housekeeping genes) for relative quantification (qPCR $\Delta\Delta C_T$). The asterisks indicate the primers finally used in the experiment for the quantification of AMF relative to the total amount of fungi.

	Primer pairs	Target gene	Reference
**AMF specific**	AMG1F/AM1*	18S rRNA	[37]
**Endogenous control**	ITS1F/ITS2*	ITS1 region	[66]
	GAP_f1/GAP_r3	GAPDH	[37]
	GH63IF/GH63IR	GH63	[67]
	SPRYpF/SPRYpR	SPRYp	[68]
	Vps26F/Vps26R	Vps26	[68]
	RasF/RasR	Ras	[68]
	T1/Bt2b	β-tubulin	[68]
	Bt2a/Bt2b	β-tubulin	[68]

Table S3. Truffle-oak's growth variables. Treatment effect on truffle-oaks growing alone and co-cultured with medicinal aromatic plants either inoculated or non-inoculated with arbuscular

mycorrhizal fungi (AMF). Variables were log (∞) transformed when necessary to meet the requirements to perform the ANOVA analysis (ANCOVA in the case of shoot dry weight), and significant values are highlighted in bold ($P < 0.05$). LMA=Leaf Mass per Area.

Truffle-oak variables	term	df	Sum sq	Mean sq	F-statistic	p-value
Shoots dry weight$^{\infty}$	treatment	6	1.715	0.286	18.704	**<0.0001**
	initial height	1	0.100	0.100	6.574	**0.0141**
	Residuals	41	0.626	0.015		
Roots dry weight$^{\infty}$	treatment	6	0.258	0.043	1.354	0.2554
	Residuals	42	1.336	0.032		
Shoot to root ratio$^{\infty}$	treatment	6	2.507	0.418	9.215	**<0.0001**
	Residuals	42	1.905	0.045		
Height$^{\infty}$	treatment	6	0.136	0.023	5.064	**0.0005**
	Residuals	42	0.188	0.004		
Diameter$^{\infty}$	treatment	6	0.102	0.017	3.442	**0.0074**
	Residuals	42	0.208	0.005		
LMA	treatment	6	6.858	1.143	3.962	**0.0031**
	Residuals	42	12.118	0.289		
Chlorophyll content	treatment	6	141.280	23.547	0.503	0.8023
	Residuals	42	1965.141	46.789		

Table S4. Medicinal and aromatic plants´ (MAPs) growth variables. Effect of arbuscular mycorrhizal fungi (AMF) on inoculated *vs* non-inoculated MAPs co-cultured with truffle-oaks, analysed by ANOVA; significant values are highlighted in bold ($P < 0.05$).

Species	MAPs variables	term	df	Sum sq	Mean sq	F-statistic	p-value
**Lavender**	Shoots dry weight	AMF	1	231.227	231.227	32.116	**0.0001**
		Residuals	12	86.397	7.200		
	Roots dry weight	AMF	1	23.049	23.049	4.294	0.0605
		Residuals	12	64.418	5.368		
	Shoot to root ratio	AMF	1	0.805	0.805	1.682	0.2190
		Residuals	12	5.744	0.479		
	Height	AMF	1	58.018	58.018	2.244	0.1600
		Residuals	12	310.230	25.853		
	Lavender spikes	AMF	1	42.875	42.875	43.713	**0.0000**
		Residuals	12	11.770	0.981		
**Sage**	Shoots dry weight	AMF	1	104.261	104.261	11.801	**0.0049**
		Residuals	12	106.016	8.835		
	Roots dry weight	AMF	1	82.889	82.889	10.693	**0.0067**
		Residuals	12	93.021	7.752		
	Shoot to root ratio	AMF	1	0.131	0.131	1.378	0.2633
		Residuals	12	1.142	0.095		
	Height	AMF	1	103.143	103.143	9.691	**0.0090**
		Residuals	12	127.714	10.643		
**Thyme**	Shoots dry weight	AMF	1	24.314	24.314	5.004	**0.0450**
		Residuals	12	58.307	4.859		
	Roots dry weight	AMF	1	26.714	26.714	3.125	0.1025
		Residuals	12	102.598	8.550		
	Shoot to root ratio	AMF	1	0.090	0.090	0.201	0.6618
		Residuals	12	5.368	0.447		
	Height	AMF	1	1.341	1.341	2.036	0.1791
		Residuals	12	7.905	0.659		

Table S5. Quantification of arbuscular mycorrhizal (AM) extraradical mycelium in soils with truffle-oak growing together with AM lavender, sage or thyme. AM mycelium quantification is relative to each species´ control i.e., AM-lavender, AM-sage or AM-thyme growing alone, in which the quantity of AMF was considered as 100. Data are means ± SE of relative gene expression and were calculated by $\Delta\Delta C_T$ method.

Treatment	Relative quantification of AMF
truffle-oak + AM-lavender	36.2 ± 9.5
truffle-oak + AM-sage	36.4 ± 6.1
truffle-oak + AM-thyme	33.4 ± 9.6

Table
S6. Percentage of arbuscular mycorrhizas (AM) in roots of medicinal and aromatic plants (MAPs) growing alone or with truffle-oaks. Data are means ± SE.

Treatment	AMF mycorrhizas (%)
AM-MAPs	57 ± 5.1
truffle-oak + AM-MAPs	65.6 ± 2.5

Figure S3. *Quercus ilex* roots colonized by a) *Tuber melanosporum* and b) arbuscular mycorrhizal fungi, from co-cultured plants collected at the end of the experiment.

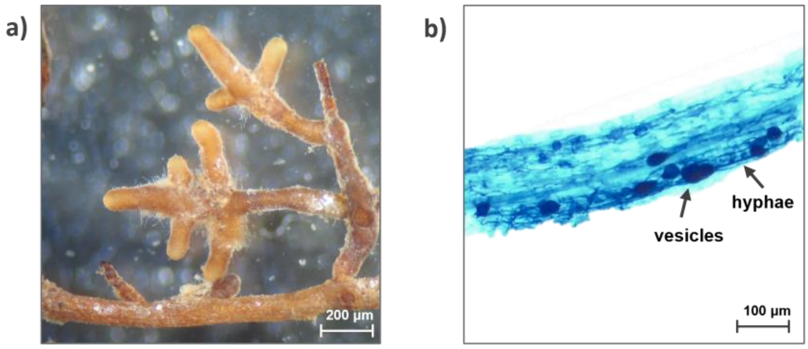

References

37. Bodenhausen, N.; Deslandes-Hérold, G.; Waelchli, J.; Held, A.; van der Heijden, M.G.A.; Schlaeppi, K. Relative qPCR to Quantify Colonization of Plant Roots by Arbuscular Mycorrhizal Fungi. *Mycorrhiza* **2021**, *31*, 137–148, doi:10.1007/s00572-020-01014-1.

66. Toju, H.; Tanabe, A.S.; Yamamoto, S.; Sato, H. High-Coverage ITS Primers for the DNA-Based Identification of Ascomycetes and Basidiomycetes in Environmental Samples. *PLoS One* **2012**, *7*, e40863, doi:10.1371/journal.pone.0040863.

67. Pérez-Izquierdo, L.; Morin, E.; Maurice, J.P.; Martin, F.; Rincón, A.; Buée, M. A New Promising Phylogenetic Marker to Study the Diversity of Fungal Communities: The *Glycoside Hydrolase* 63 Gene. *Mol Ecol Res* **2017**, *17*, e1–e11, doi:10.1111/1755-0998.12678.

68. Tao, Y.; van Peer, A.F.; Huang, Q.; Shao, Y.; Zhang, L.; Xie, B.; Jiang, Y.; Zhu, J.; Xie, B. Identification of Novel and Robust Internal Control Genes from *Volvariella volvacea* That Are Suitable for RT-QPCR in Filamentous Fungi. *Sci Rep* **2016**, *6*, 29236, doi:10.1038/srep29236.
